# The acceptability and impact of a randomised controlled trial of welfare rights advice accessed via primary health care: qualitative study

**DOI:** 10.1186/1471-2458-6-163

**Published:** 2006-06-21

**Authors:** Suzanne Moffatt, Joan Mackintosh, Martin White, Denise Howel, Adam Sandell

**Affiliations:** 1 Public Health Research Group, School of Population and Health Sciences, Faculty of Medical Sciences, University of Newcastle upon Tyne, NE2 4HH, UK

## Abstract

**Background:**

Qualitative research is increasingly used alongside randomised controlled trials (RCTs) to study a range of factors including participants' experiences of a trial. The need for a sound evidence base within public health will increase the need for RCTs of non-clinical interventions. Welfare rights advice has been proposed as an intervention with potential to reduce health inequalities. This qualitative study, nested within an RCT of the impact of welfare rights advice, examined the acceptability of the intervention, the acceptability of the research process and the perceived impact of the intervention.

**Methods:**

25 men and women aged 60 years or over were recruited from four general practices in Newcastle upon Tyne (UK), a sub-sample of those who consented to be contacted (n = 96) during the RCT baseline interview. Semi-structured interviews were undertaken and analysed using the Framework Method.

**Results:**

Participants viewed the trial positively although, despite agreeing that the information leaflet was clear, some had agreed to participate without being fully aware of what was involved. Some participants were unaware of the implications of randomisation. Most thought it fair, but a few concerns were raised about the control condition. The intervention was acceptable and made participants feel confident about applying for benefit entitlements. 14 out of 25 participants received some financial award; median weekly income gain was £57 (€84, $101). The perceived impact of additional finances was considerable and included: increased affordability of necessities and occasional expenses; increased capacity to deal with emergencies; and a reduction in stress related to financial worries. Overall, perceived independence and ability to participate in society increased. Most participants perceived benefits to their mental well-being, but no-one reported an improvement in physical health. The RCT showed little or no effect on a wide range of outcome measures.

**Conclusion:**

Participation in the trial and the intervention was acceptable to participants. Welfare rights advice targeted at people aged 60 years or over and accessed via primary care had a positive impact on quality of life and resulted in increased social participation. The divergence of qualitative and quantitative findings suggests that both methods make important contributions to the evaluation of complex social interventions.

## Background

Qualitative research is increasingly used alongside randomised controlled trials (RCTs) to study factors such as recruitment[1], participants' experiences[2], lay understandings of randomisation[3] and informed consent[4], and to illuminate reasons why the intervention may or may not have worked[5]. Much of this work has focused on participant experiences in clinical trials, but the need for a sound evidence base for public health[6] will increase the need for RCTs of non-clinical interventions. While evidence of outcomes can be derived from RCTs, 'evidence of the process by which those outcomes were achieved, the quality of implementation of the intervention, and the context in which it occurred is likely to come from qualitative data'[7] (p528).

Welfare rights advice can lead to significant financial and non-financial gains [8-10], and it has been proposed as an intervention with significant potential to reduce inequalities in health[11]. Previous qualitative research has indicated that welfare rights advice accessed via primary care is viewed positively and is perceived to address both social and health needs[12]. A number of studies of welfare rights advice accessed via primary care indicate the usefulness of such services and their perceived acceptability among recipients, primary care and welfare rights staff [13-17]. However, no robust qualitative work has examined the acceptability and health impact of this social intervention within the context of an RCT among those aged 60 years or over.

In this qualitative study, undertaken within the context of a pilot RCT reported in an accompanying paper[18], we aimed to assess: the acceptability of a domiciliary welfare rights advice service accessed via primary care; the acceptability of the research process; and the perceived impact of the intervention, in order to inform future studies.

## Methods

The study took place in parallel with a single blind pilot randomised controlled trial with allocation of individuals to intervention (receipt of welfare rights advice immediately) or control condition (receipt of intervention after a six month delay), the methods of which are described in detail in the accompanying paper[18]. The intervention comprised a domiciliary assessment of current welfare status and benefits entitlement, active assistance in making claims where appropriate, and follow-up for unresolved claims as required.

**Figure 1:**
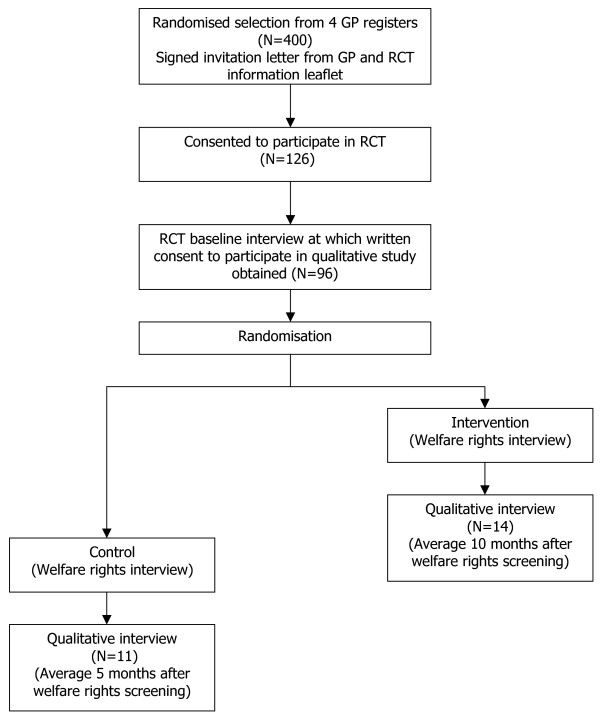
Flow chart showing sequence of participant selection for qualitative study.

Participants were selected using general practice databases and the selection process is summarised in Figure 1. A random sample of patients aged 60 years or over from each of four participating practices in Newcastle upon Tyne (UK) was invited to take part in the trial. The invitation comprised a covering letter signed by the GP together with a leaflet (see table 1) explaining the intervention and the RCT. Those who consented to participate were interviewed with a structured baseline assessment, at which time they were asked if they would be willing to take part in a further qualitative study for which written informed consent was obtained. The sampling frame for the qualitative study was formed by those (n = 96) who consented to be approached for the qualitative study during the RCT baseline interview. All participants were then randomised into intervention and control groups. The intervention (welfare rights assessment interview) took place approximately two weeks after the baseline assessment for the intervention group and approximately six months after the baseline assessment for the control group. The qualitative study sample comprised respondents from intervention and control groups, purposively selected to include those eligible for the following resources: financial only; non-financial only; both financial and non financial; and none. Sampling continued until no new themes emerged from the data[19].

Appointments were arranged by telephone and semi-structured interviews were undertaken by SM between April and December 2003 in participants' homes. The average time between the welfare rights consultation and research interview was ten months for the intervention group and five months for the control group because the control group received their welfare rights assessment six months after the intervention group. The topic guide covered perceptions of the impact of material and/or financial benefits on physical/mental health, health related behaviours, social benefits and the acceptability of the intervention and research process. Interviews were audio-recorded and transcribed in full.

We undertook thematic analysis following the Framework method,[20] with constant comparison[21] and deviant case analysis[22] to enhance internal validity. Resulting typologies were derived and higher level descriptive and explanatory categories developed.

### Ethical approval

The protocol was approved by the Newcastle upon Tyne joint universities and NHS research ethics committee.

## Results

### Participants and benefits received

Twenty-five semi-structured, in-depth interviews were undertaken (14 intervention, 11 control). Ten participants were interviewed with partners who made active contributions. Fourteen participants received some financial award. The median weekly income gain was £57 (€84, $101) (range £10 (€15, $18) to £100 (€148, $178)) representing a 4–55% increase in household income. Eighteen participants were in receipt of benefits, either as a result of the current intervention or because of claims made prior to the study. Although 96 of the total sample (126) agreed to participate, data saturation was reached after interviewing 25 respondents.

### Acceptability and understanding of the research process

Participation in the RCT required considerable commitment. All participants were interviewed four times over 24 months with the same structured interview schedule and the qualitative sub-sample participated in a further in-depth interview. Participation rates for the RCT remained high (87%) after two years (five deaths; one moved, 11 declined follow-up).

The research process was a positive experience overall (table 2). Factors which contributed to this included: enjoying the company of others; learning through participation; the domiciliary nature of the intervention and research; trust in the institutions involved (NHS and University); and building a relationship with the researchers. No one was concerned about the time commitment, divulging personal information or breaches of confidentiality.

During the qualitative interview, participants re-read and commented on the participant information leaflet. There was unanimous agreement that it was clear, straightforward and also reflected their experiences of being in the study. However, it became apparent that a number of people had agreed to take part in the study without being fully aware of what it entailed. Some participants disclosed that they had not realised there was the potential for gaining additional resources, despite this being clearly stated during invitation and consent. The main reasons given for taking part were: altruism – helping others and highly regarded institutions like the NHS; importance of research for generating new knowledge; and, finding out about welfare benefit entitlements (table 3). Their GP's signature on the invitation letter was another reason given for participation, as it conferred a degree of legitimacy on the research. Participants also felt they were reciprocating for the care they had received from their GPs, whose involvement in the research, they felt, signified a caring attitude.

Although the randomisation procedure was fully explained, some participants were not aware of its implications and a number were unaware to which group they were randomised (table 4). However, when the procedure was explained, most participants, whether in the intervention or control group, thought that randomisation was fair. Two participants did voice reservations about the control condition on the basis that this resulted in a denial of income.

### Views on the intervention

The intervention was regarded positively by participants, irrespective of whether they gained personally (table 5). The reasons for this were linked to the fact that this particular welfare rights service did not operate in a conventional way. The assessment was offered rather than sought and any claims arising were as a result of a knowledgeable professional actively assisting respondents to make claims. This effectively sanctioned claims as well as lessening the chances of rejection. Providing the service in the respondent's home meant that it was convenient, safe and particularly accessible for those with sensory or mobility problems. The most common description of the service was 'helpful', but many participants spoke at length about having the chance to talk, and having someone take an interest, suggesting that the welfare consultation itself had a therapeutic effect. Among those who did not qualify for benefits, there was no evidence that their hopes had been raised and dashed by no subsequent claim being made.

The process of the intervention required the disclosure of a considerable amount of information about personal finances. No-one objected to this in the context of the welfare rights assessment, although one participant did not wish to disclose actual amounts. Most participants saw this as a necessary part of the process, and many were already required to disclose finances for other matters, such as qualifying for council tax rebates. Participants highlighted the fact that the service made them feel relaxed, at ease and confident about applying for benefits.

### Impact of the intervention

**Figure 2:**
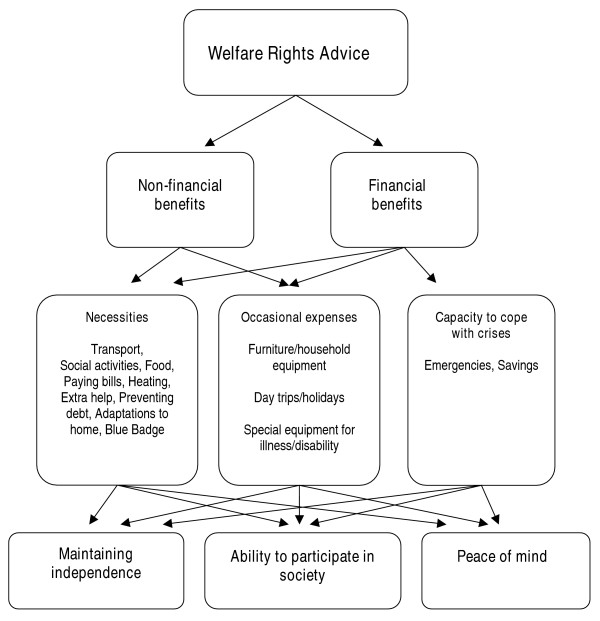
Conceptual model of reported impact of gaining additional financial and non-financial resources.

Fourteen participants received additional financial resources. The perceived impact of extra money was considerable and it was used on a wide range of items. From participants' accounts, four linked categories were identified (table 6). Firstly, increased affordability of *necessities*, without which maintaining independence and participating in daily life was difficult. This included accessing transport, maintaining social networks and social activities, buying better quality food, paying bills, preventing debt and affording paid help for household activities. Secondly, *occasional expenses *such as clothes, household equipment, furniture and holidays were more affordable. Thirdly, extra income was used as a means of dealing with potential *emergencies *and to increase *savings*. Fourthly, all participants described the easing of financial worries as bringing '*peace of mind'*. Figure 2 summarises the ways in which these categories may be linked.

## Discussion

### Main findings and interpretation

Participation in this RCT was regarded as a positive experience by most interviewees; the intervention was highly regarded and had wide-ranging impacts, particularly for those who gained new benefits.

Participants' perspectives in RCTs have generally been measured by using attitude questionnaires with a view to improving participation rates[3]. Although limited by the questionnaire format, such studies show that the main reasons given for anticipated participation in trials were altruism[23]and personal benefit[24], and major disadvantages identified were time involved and problems with travel[25,26]. Altruism, generating new knowledge through research and personal benefit were the main reasons given for participation in this study. Because of its domiciliary design, the study did not require participants to travel, something that many, particularly those with limited mobility or sensory impairments, appreciated and which may have increased recruitment and reduced attrition. Our study also suggests that the involvement of a trusted health professional in the recruitment can increase participation rates.

Although every effort was made to ensure that consent was fully informed, it became clear that a number of people did not understand the nature of the intervention or were not fully aware that they were being randomised. This occurred despite ethical committee approved information leaflets that were regarded as clear and a good reflection of participants' experiences in the trial. This has also been found in clinical research[4]. With the exception of two participants, no-one was unhappy about randomisation to the control condition which involved waiting six months for welfare rights advice. Participants rationalised this in three main ways: firstly that it was a fair way to 'ration' a service that was otherwise not usually available; secondly, that people must face the consequences of their informed consent; and, thirdly, a degree of fatalism and/or ignorance about group allocation. Participants in this study appeared to have a more relaxed attitude to randomisation than has been reported in clinical trials[3]. This could be explained by differences in the nature and context of clinical and social interventions, the former concerning treatment for an existing condition with anticipated health benefits; the latter, as in this case, concerning access to a potentially valuable service, but with unknown health effects. This study demonstrates that it was acceptable to randomise the intervention with a delay of six months to the control group. However, the pilot RCT findings suggest that this is unlikely to be adequate time for any measurable health benefits to become evident [18]. A longer delay between the study and control group's receipt of the intervention raises ethical issues that require further consideration [27].

### Strengths and limitations

The qualitative data were derived from a sub-sample of the RCT participants and it is possible that only those willing to be interviewed further held such positive views. Comparison between the quantitative and qualitative samples on a number of social and economic variables indicated similarities according to age, proportion of those living alone/as a couple, council tax band and long term limiting illness, suggesting that both samples were not qualitatively different [18,28].

## Conclusion

The effects of the intervention were wide-ranging and positively regarded by participants. However, this was not mirrored by the outcome measures used in the pilot RCT. Despite measuring a wide range of physical, psychological and social outcomes, the pilot RCT found little or no differences between intervention and control groups, or between those who did and did not receive additional resources suggesting that the intervention had no impact on these outcome measures [18,29]. It is not uncommon for qualitative and quantitative studies to produce divergent findings [2,30,31] and it is likely that each method, with its different epistemological underpinnings, captured different aspects of phenomena under investigation. The qualitative approach enabled participants to give an account of the various ways in which the intervention impacted on their lives, such as increased independence and improved quality of life, which were not explicitly measured in the pilot RCT and are challenging to capture quantitatively[29]. This qualitative study has thus not only provided valuable information about outcome measures for a future trial, but helped with the overall interpretation of the pilot RCT. The study also suggests that inclusion of a qualitative component will help to illuminate the process and outcome of a future trial.

## Competing interests

The author(s) declare that we have no competing interests.

## Authors' contributions

SM and MW with DH and AS initiated the RCT and obtained funding. SM designed the qualitative study, undertook the interviews, analysis and initial interpretation. JM assisted with devising the coding framework. SM, JM, MW, DH and AS jointly undertook final interpretation of the data. SM wrote the first draft of this paper, JM, MW, DH and AS commented on subsequent drafts. All authors approved the final version. All authors are guarantors and accept full responsibility for the conduct of the study and the contents of this paper.

## Pre-publication history

The pre-publication history for this paper can be accessed here:



## Figures and Tables

**Table 1:** Extracts from participant information leaflet

**What is this study about?**
A lot of people do not get the state welfare benefits that they should. We are testing out a new way of giving advice about welfare rights in your doctor's surgery. We are trying to find out how this advice may help and whether there are any health benefits. We are inviting you to take part in this study.

**What does this involve?**
Mainly, we need you to give up a little of your time. You will be asked some questions about your health and circumstances in a private interview. This will probably take about an hour. After this you will be given an appointment to see a Welfare Rights Officer from Newcastle Social Services, who will give you confidential advice about your rights to state and other benefits, and help with making claims if appropriate. If you have difficulty filling out forms, help will be provided. The Welfare Officer can come to your home or meet with you at your general practice.

In this study, one group of people will be given an appointment with the welfare advice service straight away and another group of people will be given an appointment a few months later. The group you are put in will be decided by chance, like tossing a coin. However, everyone will get an appointment within 6 months.

**Table 2:** Experiences of the research process

I feel that I'm enjoying it, I mean I've quite enjoyed listening and as I say, it's benefited us in more ways than one, because it's given me ideas and possibly it's given you ideas as well. (Case 8, male, 77)

I was apprehensive about going out, I thought maybe I had to go to a hospital meeting, and I found it wonderful, it's all in my own home. (Case 3, female, 72)

You look at the stature of the organisation involved and the people that come to see you. (Case 20, male, 72)

Some of the questions have been personal but there has been no sort of awkwardness and everything they have asked me I have answered ... and they have been very friendly ... there has been no sort of dictatorial attitude or anything. (Case 1, male, 75)

Basically, when someone comes and says, well it's totally confidential, I believe them that it is totally confidential. I mean I understand the Data Protection Act ... so you know, no problem whatsoever. (Case 18, male, 65)

**Table 3:** Motivation for taking part

I feel it's sort of putting something back. (Case 22, female, 78, increased Attendance Allowance from low to high rate and Staywarm)

I didn't realise I was claiming for money ... it wasn't put to me like that. It was just a study, people were coming to ask you questions and you would answer them ... I didn't realise at all about getting a grant. (Case 1, male, 75, gained higher rate Attendance Allowance)

Well, if we don't take part in research we're never going to learn ... research produces information that you can properly base your next decision on. (Case 20, male, 72, no gain)

Well, there's no harm in anybody coming to the house and anybody asking questions. That's the way I look at it and if it's going to benefit you, why shouldn't you accept them. (Case 4, male, 74, gained Attendance Allowance (lower rate) and Invalid Care Allowance)

Well they are reliable, sensible, you know, educated people, and they know what they are talking about. And if they go to the trouble of writing me a letter, well it must be for some good. (Case 25, female, 82, no gain)

Coming from your doctor, you know ... obviously they're not going to ask you if it's not important. (Case 10, male, 65, pension credit)

**Table 4:** Views on randomisation

I probably wouldn't know whether I was in the first group or the second group, so I mean you wouldn't be worried about it ... . Well I don't think it would bother me really because you got there in the end you know. It's not like it's a race ... it's just one of those things isn't it. (Case 19, female, 82, intervention)

If they couldn't see everyone all at once ... if you say it was randomly done, so they didn't even assess the pros and cons of the person's case so that was fair, wasn't it? (Case 7, male, 75, control)

I mean, if people sign up and they know that they're on course to be seen six months later, and if they're unfortunate to be in the second group, hard cheese. (Case 8, male, 77, intervention)

I thought, well that would have been lovely if I'd had that six months ago or a year ago. I mean I didn't know that you'd done what you'd said you'd done. (Case 22, female, 78, control)

Well, I just accepted that really. I thought, well if I wasn't in the first group that would be it, I would just come into the second group. But as time goes on, maybe I might have thought it was unfair you know that I could have had that money and all this help sooner. (Case 2, female, 74, intervention)

**Table 5:** Views on the intervention

I found someone was interested in me and come and seen me ... that makes a big difference. It's nice to get someone to discuss things with and ... assure me that I was entitled to this ... I couldn't believe it. (Case 17, female, 84, weekly income increased by 52%)

You talk to someone like [the advice worker] you felt that in two or three minutes she knew her business ... she would give you a very well-informed and reliable answer immediately. (Case 19, female, 82, weekly income increased through another welfare rights service prior to this study)

I wouldn't have felt any animosity ... I was already prepared for the fact that I wouldn't be entitled to anything. (Case 8, male, 77, no gain)

When [welfare rights officer] asked about finances and everything, I've nothing to hide, so I just told her the truth. (Case 23, female, 74, no gain)

... other than saying I wasn't prepared to divulge what finances we had, apart from that she didn't press us. She said excess £30,000, I said yes, you could say that. (Case 12, male, 79, weekly income increased by 18%)

I'm used to taking all my papers up to the Council Office for the rent benefit and that, so, I'm not stupid, it didn't worry me. I know it does worry a lot of people, but I'm not stupid. (Case 14, female, 82, weekly income increased by 51%)

**Table 6:** Impact of additional resources

***Necessities***
I couldn't bear it [not having a car] ... just to have the car there is such a godsend and its gets you out anyway because my walking isn't so good. (Case 22, female, 78, weekly income increase £19 (€28, $34))

I am eating more fruit and things that you couldn't of bought before ... you don't need to think about, can I afford it? You can, and as I say, I buy more fruit. (Case 3, female, 72, weekly income increase £57 (€84, $101))

***Occasional expenses***
We have been quite depressed last month ... now we can afford petrol to go up the coast in the car. Tank full of petrol if we feel like it ... we cheered ourselves up ... whereas it would have been another miserable day ... it did us good. (Case 15, female, 64, weekly income increase £72 (€106, $128))

***Capacity to cope with crises***
We have always been in the position where we have never had an awful lot of cash in the bank ... if we got an emergency we have got something there for it now. (Case 7, male, 75, weekly income increase £39 (€58, $69))

***Peace of mind***
[it makes] all the difference in the world. From being worried about bills to not being worried, and to be able to keep the heating on and not bother when the bill mounts up a bit ... but now I needn't worry ... it's the best help of all really, the lack of worry about finance. The worst thing for me that depressed me and got me down more than anything was worrying about not being able to pay bills. (Case 19, female, 82, income increased through another welfare rights service prior to this study)
